# Recent Developments in the Applications of GO/rGO-Based Biosensing Platforms for Pesticide Detection

**DOI:** 10.3390/bios13040488

**Published:** 2023-04-19

**Authors:** Geetha Gopal, Namrata Roy, Amitava Mukherjee

**Affiliations:** Centre for Nanobiotechnology, Vellore Institute of Technology, Vellore 632014, India

**Keywords:** graphene oxide (GO), reduced graphene oxide (rGO), biosensors, pesticides, detection

## Abstract

Pesticides are often used in different applications, including agriculture, forestry, aquaculture, food industry, etc., for the purpose of controlling insect pests and weeds. The indiscriminate usage of pesticides poses a massive threat to food, environmental, and human health safety. Hence, the fabrication of a sensitive and reliable sensor for the detection of pesticide residues in agro products and environmental samples is a critical subject to be considered. Recently, the graphene family including graphene oxide (GO) and reduced graphene oxide (rGO) have been frequently employed in the construction of sensors owing to their biocompatibility, high surface-area-to-volume ratio, and excellent physiochemical, optical, and electrical properties. The integration of biorecognition molecules with GO/rGO nanomaterials offers a promising detection strategy with outstanding repeatability, signal intensity, and low background noise. This review focuses on the latest developments (2018 to 2022) in the different types of GO/rGO-based biosensors, such as surface plasmon resonance (SPR), fluorescence resonance energy transfer (FRET), and electrochemical-based techniques, among other, for pesticide analysis. The critical discussions on the advantages, limitations, and sensing mechanisms of emerging GO/rGO-based biosensors are also highlighted. Additionally, we explore the existing hurdles in GO/rGO-based biosensors, such as handling difficult biological samples, reducing the total cost, and so on. This review also outlines the research gaps and viewpoints for future innovations in GO/rGO-based biosensors for pesticide determination mainly in areas with insufficient resources.

## 1. Introduction

Agriculture is the primary activity for mankind, and it provides a living for around 60% of the world’s population. Many scientific techniques are required to increase the sustainability of agricultural products so that population demand can be reduced. Pesticides have become an essential element in the growth of agriculture as a plant protection agent to increase food security since these substances play a key part in preventing many deadly diseases [1]. In current metropolitan environments, pesticides are a frequent source of contamination in the soil, air, and water as well as on nontarget creatures. Hence, it is crucial to continually look out for pesticide residues in edible plant materials like leaves, fruits, and vegetables as well as any other parts that can be contaminated. This will help to prevent the ingestion of dangerously excessive levels of these toxic substances. In recent years, a number of products and technologies for pesticide detection have been created [2]. 

It is vital to create analytical methods that can detect pesticides quickly, accurately, and economically. In fact, the EU Regulation 2013/39/EU promotes the development of such methods with the need that they be sufficiently sensitive to assure that any infraction of surface water EQS limits is accurately identified and quantified. The development of novel sensing techniques for the detection of pesticides in water, soil, and food is currently the focus of many researchers. Liquid/gas chromatography (GC), high-performance liquid chromatography (HPLC), electrophoresis, and mass spectroscopy are the most frequently used sensing techniques. However, these techniques have demonstrated certain disadvantages including a site-specific and sensitivity toward target pollutants [3]. There have since been various attempts to develop new technologies for the quick, sensitive, focused, accurate, and user-friendly identification of pesticides. This review article’s goal is to propose an alternate method for effective pesticide sensing that makes use of nanotechnology. Biosensors are rapid, economical, practical, and highly sensitive devices that can identify and measure targets by converting the target’s physical recognition into optical, electrical, and magnetic signals. The use of biosensors to detect toxins in food and the environment is thought to be a promising technology [4]. Pesticide sensing values can be determined directly through the biosensors by translating the acquisition signal. Due to their super surface effect and small size effect, nanoparticles such carbon nanomaterials, semiconductor nanomaterials, polymeric nanomaterials, and metal nanomaterials have drawn considerable attention and have been connected in multiple sectors. In the development of biosensors, nanomaterials are often used as transducer elements. A biosensor is composed of four components: a bioreceptor, a transducer, a signal processor that transforms electronic signals into desired signals, and a display interface. Nanomaterial is used in a biosensor component to reduce electro-active chemical power density. Additionally, it serves to label and multiply the observed signals at various time intervals [5]. 

**Figure 1:**
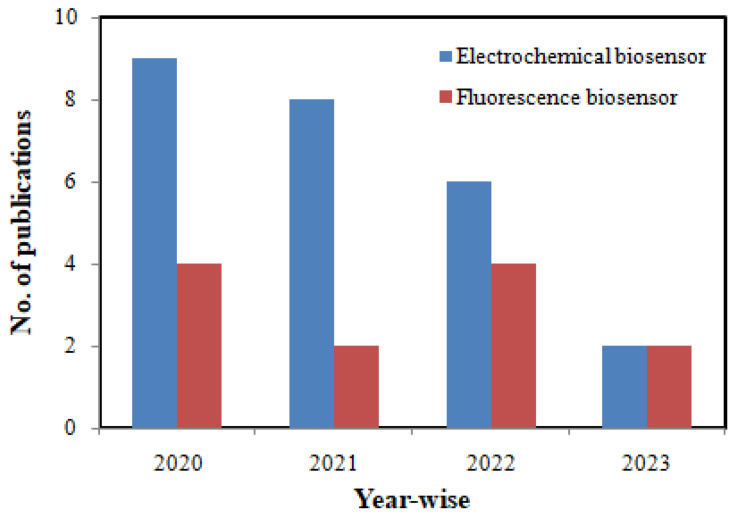
Year-wise development of GO/rGO-based biosensors for pesticide detection.

The main objective of this review article is to provide readers a brief overview of the most recent developments in GO/rGO biosensors for pesticide detection in aqueous medium. The time span for the literature survey has been restricted to the previous four years (2020–2023) only. After a brief overview of electrochemical biosensors, FRET biosensors and their capabilities and performance in environmental pesticide detection are critically assessed. We believe that the scientific fraternity involved in the study of pesticides and those with an interest in the development of GO/rGO nanomaterial-based biosensors will find this review to be useful. Future research opportunities and current obstacles are also critically discussed. The year-wise development of GO/rGO-based biosensors for pesticide detection is given in Figure 1. 

## 2. Pesticide Detection and Toxicity

**Figure 2:**
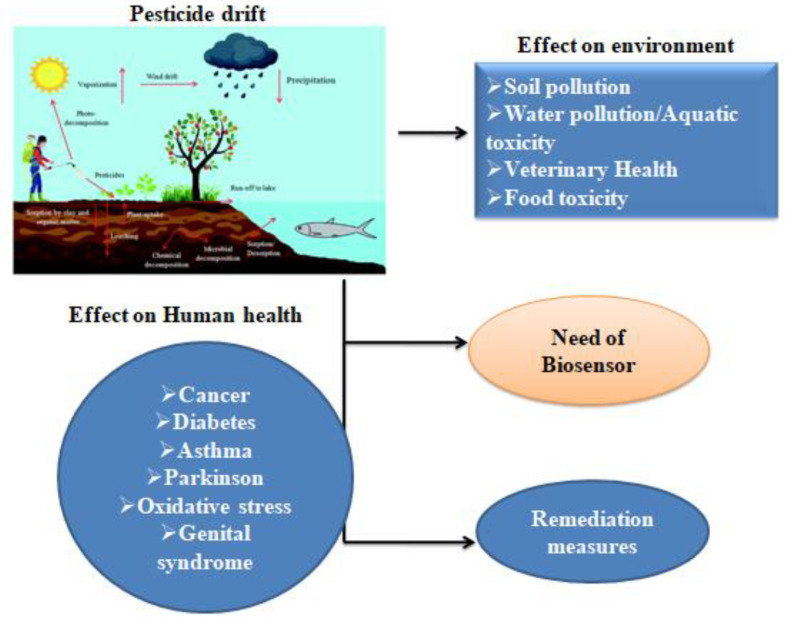
Drifting of pesticides into the environment and its toxic effect.

As per the United States Code of Federal Regulations (CFR) classification, a pesticide is any substance or combination of ingredients designed to be employed as a plant promoter, selective herbicide, or desiccant (United States Environmental Protection Agency, Washington, DC, USA, 2004). The Food and Agriculture Organization (FAO, Washington, DC, USA) defines pesticides as any compound or combination of molecules used for eradicating pests, disease-carrying animals, undesirable plant or animal species, and pests that affect food production, management, sale, storage, or transportation. As per a recent estimate, more than 4.19 million metric tonnes of pesticides were consumed globally in 2019. China was the highest consumer (1.76 million metric tonnes), followed by the Unites States (408 thousand tonnes), Brazil (377 thousand tonnes), and Argentina (204 thousand tonnes). In another alarming statistic, the WHO cautioned about an increase in pesticide use each year in Southeast Asian countries, which includes Cambodia, Laos, and Vietnam [6]. Drifting of pesticides into the environment and its toxic effect is given in Figure 2.

➢ **Effect of pesticides on ecosystems**

The fatal effect of exposure to pesticides persisting over a longer time period is referred to as chronic toxicity. As per the commonly used “WHO Recommended Categorization of Pesticides by Hazard”, pesticide residues are now grouped into “WHO Hazard classifications” [7]. The pesticide-infested surface and groundwater have affected aquatic life and human health. The bioaccumulation of pesticides in the tissues of aquatic organisms declines the biodiversity in the population, thus disturbing the food chain. Amongst the various pesticide residues, organochlorine substances (OCPs) have already been widely utilised across the world to manage agricultural pests and disease vector ailments (dengue and other disease outbreaks). The application of these compounds in an unrestrained manner seems to hold the risk of harming the environment, portable water, and health. Pesticide application offers a barrier of defence against other pests that feed on pods, but defective pods might not even produce seeds, or they might be of poor quality and unusable. Pesticide toxicity in the soil can have significant environmental and agricultural impacts. The presence of pesticides in soil has detrimental effects such as soil degradation, nontarget effects, disruption of nutrient cycling, and soil erosion via the reduction of soil stability [8].

➢ **Effect of pesticides on human health**

Continuous exposure to several OCPs over time can harm the nervous system and cause cancer, immune system abnormalities, birth defects, and reproductive problems [9]. Different pesticides are also known to cause cancer; for instance, prostate cancer is the most prevalent of all the different cancers and is linked to organophosphorus (malathion and parathion), which impacts cellular growth and proliferation [10]. There have been reports that a number of pesticides residues, including DDT, chlorpyrifos methyl, and organochlorine, can alter the epigenetic methylation sequence in humans. Pesticides can enter the body through ingestion, inhalation, or absorption through the skin, and once inside the body, they can affect different systems and organs. The pesticides can have neurotoxic effects and affect cognitive functions, leading to developmental growth delays in children. Pesticides can also function as endocrine disruptors causing hormonal imbalances that can interfere with reproductive functions. Renal failure and kidney dysfunction are major symptoms reported in the case of pesticide toxicity. The immunotoxicity precipitate autoimmune diseases, induce chronic inflammation, decrease antibody production, and alter gene expression, thus making individuals vulnerable to various diseases and infections [11]. 

According to research, pesticides can affect acetylcholinesterase (AChE) activity, which in turn can influence the neurological system and lead to a variety of neurotoxic effects (neurotoxicity) in fish. It was reported that all organophosphorus anticholinesterases might have an underlying harmful mechanism, namely the phosphorylation of AChE, which results in acetylcholine build-up, overstimulation of cholinergic receptors, and subsequent clinical indications of cholinergic toxicity [12]. The risk of abnormalities in germ cells is increased by the synergistic interaction between the pesticides pyrethroids (PYR) and organophosphates (OP). Previously, Salazar-Arredondo et al. (2008) reported on chromatin and nucleic acid damage in human spermatozoa via in vitro exposure to a combination of different organophosphorus pesticide residues such as chlorpyrifosoxon, chlorpyrifos, diazoxon, and MePO (methyl-paraoxon) [13]. 

## 3. Nanomaterials Used in Biosensing Applications

There have been significant advancements in the synthesis, processing, characterization, and potential applications of a wide range of nanoscale materials, including zero-dimensional (0D) nanoparticles (such as metallic and semiconducting nanoparticles), one-dimensional (1D) nanostructures (nanowires, nanorods, and nanotubes), and two-dimensional (2D) nanostructures, including graphene nanosheets (GNs) and transition metal dichalcogenides (TMDs). These nanoscale materials have been utilized in numerous products over the past 20 years, including light-emitting diodes, memory and communication systems, magnetic discs, solar and fuel cells, batteries, supercapacitors, and catalysts [14]. Biosensors frequently incorporate the above nanomaterials because they considerably enhance performance and enable faster, more effective, and cheaper detection. The unique optical and electrical properties of materials such as silver (Ag), gold (Au) and their different forms nanowires, nanorods, nanostars, carbon nanotubes (CNT), and MWCNTs provide a high interaction surface-to-volume ratio, good conductivity, catalytic performance, and biocompatibility. Several hybrid nanostructures have also been studied in addition to pure nanomaterials. Because of their distinct optical and electrical properties, Au nanomaterials are frequently used to develop biosensors. The material’s ability to absorb strong and distinct surface plasmon resonance signals in the visible spectrum is one of its key features. SPR is a process that occurs when metal electrons are excited by electromagnetic radiation, changing the dielectric constant (Au is sensitive to this dielectric constant) [15]. Some of the widely used nanomaterials in designing biosensors for pesticide detection and their sensitivity are outlined below.

**Figure 3:**
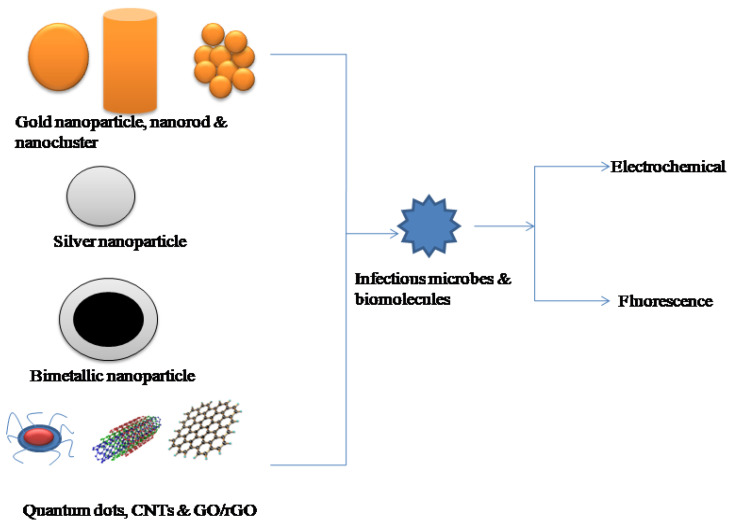
Nanomaterials used in biosensing applications.

As reported by Zhao et al., in the sensing of methomyl, AuNM–based biosensors displayed low LOD value of 81 ng L^−1^ with AChE immobilization employing the substrate mercapto methamidophos and Au nanomaterials in conjunction with a working carbon electrode [16]. Through using Au–S bonds in an electrochemical biosensor, Lin et al. were able to identify chlorpyrifos in fruit samples with a LOD of 36 ng L^−1^ [17]. Studies using AuNMs have shown strong specificity and sensitivity for chlorpyrifos, isocarbophos carbamate, and methomyl, with LOD ranging from 70 × 10^−3^ to 2.48 × 10^3^ ng L^−1^ [8]. AgNM has a wide surface ratio, elevated electron transport efficacy, and easy availability which makes it more suitable for incorporating into biosensor design. By using malathion-specific aptamers which interact with AgNPs to produce a colorimetric value, Bala et al. created an nanobiosensor with AgNPs to sense malathion in apples with a LOD of 5 × 10^4^ nM [18]. Since TiO_2_ NMs maintain catalytic activity and operate as an excellent electron donor in reactions among biomolecules and samples taking place in biosensors, they have frequently been considered to be an interface for the enzyme immobilisation of biomolecules. In order to create a TiO_2_-based sensor sol–gel carrier, Hu et al. devised a biosensor by combining carbon electrodes with TiO_2_NP and chitosan. The AChE was inhibited in order to identify dichlorvos in samples of cabbage juice as the mechanism of action. The LOD for this biosensor was 0.23 nM [19]. The different tyoes nanomaterials used in biosensing applications is given in Figure 3.

## 4. GO/rGO-Based Sensor Applications

Graphene is a hexagonal channel of covalently linked sp_2_-hybridised carbon atoms. Strong oxidants are used to treat graphite, with the addition of epoxy, hydroxyl, and carboxyl groups to its sheet-like structure. This results in the production of reduced graphene oxide (rGO). Better conductivity compared to graphene oxide, improved solvent dispersion due to the presence of functional groups, flexibility of control over rGO’s electrochemical efficiency and solubility, simplicity in manufacturing, and relatively low cost are some of the features of rGO related to better utilization in biosensor design. Additionally, the rapid transfer of electrons due to the hybridization of pz orbital electrons (sp_2_) with a short response time and lower LOD. Overall, the properties of GO and rGO are extremely valuable and include an incredibly high rigidity and tensile strength, suitability for the creation of malleable devices, excellent conductivity of electricity, good optical transparency, and low cytotoxicity; therefore, GO and rGO are likely to be used in devices that are portable [20].

**Figure 4:**
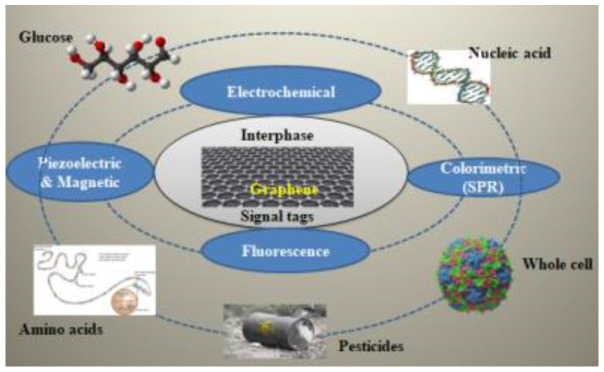
Graphene-based sensing techniques for different analytes.

In a broad sense, GO properties have been applied to numerous types of biosensors that can be roughly categorised into different types of biosensors based on (1) fluorescence resonance energy transfer (FRET), (2) laser desorption and ionization-mass spectrometry (LDI–MS), (3) surface-enhanced Raman spectroscopy (SERS), and (4) electrochemistry. Graphene-based sensing techniques for different analytes is given in Figure 4.

The significance of graphene and its derivatives as a signalling device for the identification and quantification of biomolecules, antigens, antibodies, chemicals, drugs, pesticides, DNA, whole cell viruses/bacteria, etc. lies in its suitable and excellent attributes. Chlorpyrifos and other pesticides, as well as antibiotics such as chloramphenicol, tetracycline, streptomycin, and kanamycin, can be detected using it in the environmental field. Through incorporating nanoparticles and graphene sheets, the biosensor’s sensitivity, limit of detection (LOD), and reproducibility can all be enhanced [21]. An Fe_3_O_4_–GO nanocomposite was successfully used as both the support and the magnetic core in the synthesis of a novel magnetic copper-based MOF (M–MOF–199). Five triazole pesticides were removed by magnetic dispersive solid-phase extraction from samples of tap and well water using M-MOF-199, which was characterized and employed as a sorbent. A simple, fast, and sensitive triazole pesticide detection approach from an aqueous system was established using HPLC–MS/MS and M-MOF-199 [22]. Due to the synergistic effect of the Fe_3_O_4_, TiO_2_, and rGO sheets, it was discovered that the FTG nanocomposite displayed improved detection and degrading activity against atrazine when compared to the Fe_3_O_4_/rGO, TiO_2_/rGO, Fe_3_O_4_, TiO_2_, and rGO in the aqueous system [23].

### GO/rGO-Based Nanocomposite Sensor for Pesticide Detection

The electrochemical sensing of pesticides such as methyl parathion (MP) was developed by Gao et al. with the help of graphene-based nanocomposite Au-ZrO_2_/GNs/GCE electrodes. The combined effects of Au-ZrO_2_ metal oxide and graphene nanosheets help in enhancing the electrocatalytic function of the electrode. The affinity of ZrO_2_ for phosphoric groups improves the detection of organophosphorus pesticides such as MP. Due to the presence of ZrO_2_ over the electrode surface, the chelation of phosphate groups and ZrO_2_ could increase the adsorption of MP. This type of composite electrode is capable of improving the electron transfer rate, thus causing a shift in the oxidation potential of MP. The presence of a large surface area of graphene and higher electron mobility resulting from the accelerated conductivity of the electrode assist in the accurate sensing for the direct analysis of MP [24]. Guler et al. designed an rGO-based biosensor in which AChE was immobilized using Ag@rGO/NH_2_ with the help of a crosslinking agent and glutaraldehyde and nafion serving as a protective membrane, showing excellent electrocatalytic activity. The presence of silver nanoparticles (AgNPs) increased the conductivity of the biosensor whereas the immobilization of AChE showed a decrease in Nyquist analysis, indicating a decrease in the conductivity of the biosensor. The high thickness of the AChE layer, which prevents electron transport to the working electrode surface, can be attributed to the current response tapering off when the AChE concentration was increased. Therefore, 2.56 U mL^−1^ was chosen as the optimum enzyme concentration for the following experiments. The resistance of electron transfer increased as a result of interface expansion due to the immobilization of the NA/Ag-rGO-NH_2_/GCE electrode with the AChE enzyme. The highest sensitivity recorded was 6–77 ng.L^−1^ of malathion [25]. Shanmugam et al. synthesized N, S-co-doped rGO-supported SnS_2_ nanosheets for the electrochemical-based detection of MP. The SnS_2_/N, S-rGO sensor showed a fast current response as a result of the good electrocatalytic activity and the LOD of 0.17 nM [26]. An amperometry probe was fabricated using a FeVO_4_/rGO nanocomposite modified on a glassy carbon electrode (GCE) for the sensitive detection of MP in green beans. The amperometry technique exhibited an LOD value of 0.70 nM, with practical application being demonstrated in real water samples. The evaluation of charge transfer resistance (Rct) measured using electrochemical impedance spectroscopy (EIS) showed a decrease in the Rct of the modified GCE as compared to the unmodified electrode. This indicated the easy and rapid mobility of electrons at the junction of the electrode–electrolyte interaction at the time of the oxidation reduction of the [Fe(CN)_6_]^3−/4−^ [27].

The characterization techniques involved in analysing the advanced sensing prototypes to validate and screen for high sensitivity, simple operating procedure, portability, and on-site application is a crucial aspect. In this section, we discuss cyclic voltammetry, anodic stripping voltammetry, and electrochemical impedance spectroscopy (EIS) [28]. Primarily, the dominant factor of mass transport controls the generation of current in a voltammetry via applied potential in an electrochemical sensor. The progressive change is observed in the applied potential that is independent of the time (variable), whereas the current response is linearly dependent on the analyte concentration in the test sample [29]. In recent detection strategies, voltammetry has been combined with differential pulse, anodic stripping, and square wave techniques to detect various analytes [30]. EIS is a well-known technique for the determination of impedance, and the elements of frequency-conditioned resistance and electrode capacitance are moderated by changing the current mode in alternating manner. The outcomes of the process are mathematically evaluated with a circuit (equivalent electrical circuit) which provides electrochemical mass transfer data based on quantitative analysis. Therefore, the reaction rates, conductivity, direct charge mobility (electron), and dielectric constant are variables important for the EIS technique [31]. Manavalan et al. designed a functionalized-GO based electrode sensor containing ZnO nanostars to detect the MP (methyl parathion) pesticide. The electrode was evaluated using EIS for MP sensing, and LOD of 1.2 nM and a high sensitivity of 16 µA µM^−1^ cm were recorded S [32]. Another common electrochemical technique for analysing sensors is the cyclic voltammeter (CV), which measures the cell current response as a function of applied potential in a cyclic manner. The structure of cyclic voltammograms can reveal information for the investigation of pesticides regarding the sort of working electrode reaction, the number of electrons participating in the electrochemical reaction, and the possibility of additional processes, such as adsorption or associated chemical reactions [33]. The electrochemical behaviour of the sensor, as well as the interactions between the sensor and the analyte, thus provides information on the redox reactions. Furthermore, optimization of parameters such as electrode surface area and analyte concentration contributes to the sensitivity and selectivity of the sensor material. Fu et al. fabricated an rGO-CuNP aptamer sensor for the electrochemical sensing of pesticide (profenofos, phorate, isocarbophos, and omethoate). With a cyclic voltameter (CV) in [Fe(CN)_6_]^3−^/^4−^ solution, the highest EC signal was recorded at the seventh CV cycle after which there was no increase in the signals under conditions of 0.6 mg.mL^−1^ of GO-Apt and 1.0 mM of EDTA-Cu [34].

## 5. Sensing Strategies

### 5.1. Electrochemical Sensing

**Figure 5:**
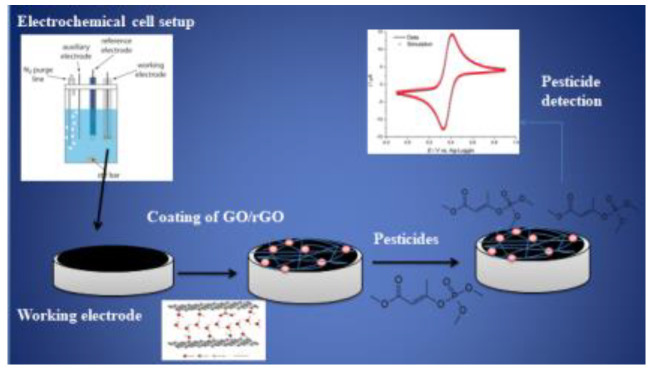
GO/rGO for the electrochemical sensing of pesticides.

Due to their numerous distinctive qualities, graphene-based nanomaterials are currently frequently employed to develop highly efficient and relatively affordable electrochemical sensors. The two main factors that contribute to graphene’s increased electrochemical activity are (1) the large surface area of its 2D sheets, which gives it a large number of electroactive sites for recognizing target molecules and increasing sensitivity, and (2) its stability over a wide temperature range, which makes it a highly reliable conductive material for the advancement of electrochemical sensors. Furthermore, the faster electron transfer kinetics of sp_2_-hybridized p_z_ orbital electrons lead to shorter response times with lower detection limits. GO/rGO for the electrochemical sensing of pesticides is given in Figure 5.

The redox behaviour of the target pesticide with the working electrode material over the applied potential range is a key factor in the electrochemical detection of pesticides. Due to the presence of “π–π” stacking and electrostatic interaction, which facilitate rapid absorbance of a variety of compounds due to synergetic effects in composites, graphene-based nanocomposites are promising materials for electrochemical sensor applications. Enzymes, antibodies, and DNA can be immobilised to produce a biosensing platform due to the large surface area. Particularly, much research has been conducted on electrochemical biosensors based on the catalytic enzyme acetylcholinesterase (AChE), which is found in the central nervous system. A comprehensive summary of the research on graphene-based bio-functionalized nanocomposites used as electrode materials for electrochemical pesticide detection is given in Table 1.

The network-like structure of the 3DG-CuO NFs nanocomposites improves the effective specific surface area and creates a suitable microenvironment for AChE loading, which could enhance the performance of the biosensor for malathion detection. The AChE-CS/3DG-CuO NFs/GCE biosensor exhibits advantages such as a broad linear relationship to malathion extending from 1 ppt to 15.555 ppb under ideal detection conditions (3 pM–46.665 nM) [35]. According to Jianhua et al., adding chitosan to graphene (Gra) will increase its mechanical flexibility while also enhancing the biosensor’s stability and detection accuracy. In one study, the surface of a glassy carbon electrode was progressively drip-coated with Gra nanofragments modified with CS and AChE using a layer-by-layer construction technique. The limit of detection for the sensor used to detect dichlorvos was 54 pM, with a concentration range of 0.1–100,000 nM [36]. Hossein et al. prepared a novel biosensor based on vanadium-disulfide-quantum-dot-doped graphene nanoplatelets/carboxylated MWCNTs on a carbon electrode. With LODs of 1.1 × 10^−14^ and 2.0 × 10^−15^ mol L^−1^ for the DPV and EIS approaches, respectively, the devised electrochemical aptasensing strategy produced a highly sensitive quantitative detection of DZN. The analytical method was also used to determine the presence of DZN in human serum, and Zayandeh Rood river water, soil, apple, and lettuce samples. The recovery rate for this method, utilizing the GCE/VS2QDs-GNP/CMWCNTs/DZBA/BSA aptasensing technique, ranged from 97.0% to 107.0% [30]. In Lijun et al.’s study, MoTe_2_ NPs/RGO heterostructures with a suitable Schottky barrier were produced using a one-step hydrothermal synthesis to improve the photoelectric performance of MoTe_2_. A label-free photoelectrochemical aptasensor for the detection of profenofos was successfully built using MoTe_2_ nanoparticles/RGO, demonstrating the material’s high visible light responsiveness and potential as a light-responsive photoactive material for biosensors. A wide linear range (10^−9^ g L^−1^ and 10^−2^ g L^−1^) and a comparatively low detection limit (3.3 × 10^−10^ g L^−1^) were both characteristics of this aptasensor [37]. Teerapat et al. reported designing CuInS_2_ microspheres with rGO able to detect chlorpyrifos in vegetable with a detection limit of 0.023 ng mL^−1^ [38].

### 5.2. Fluorescence Sensing

Both graphene and GO have the ability to quench fluorescence, which has provoked interest in their potential use in clinical and environmental research. Owing to graphene’s remarkable ability for fluorescence resonance energy transfer, it is used in fluorescence detection (FRET). It was found that GO has the maximum quenching ability, rapid binding with DNA molecules, repeatability, and selectivity to various DNA sequences when compared to nanomaterials including gold nanoparticles and carbon nanotubes. The amphiphilicity of graphene oxide enables biomolecules to adsorb on its flat surface. If the adsorbed molecule is paired with a fluorescent dye, it has the potential to conduct appropriate energy transfer, leading to fluorescence quenching with a minimal background signal [42]. At a detection limit of 5.73 nM, acetamiprid can be detected with high selectivity and minimal interference. Only one strand of dsDNA, two strands of ssDNA, and nonmodified GO, which is enzyme-and label-free, are present in the test system. More significantly, the new technique shows strong potential for use in the sectors of environmental monitoring and food regulation [43]. The development of biosensor based on rGQDs, a diazinon–specific aptamer, and MWCNTs able to detect diazinon with the detection limit of 0.4 nM (0.1 μg/L) and a linear range of 4–31 nM was previously reported [44]. A new aptasensor for the detection of diazinon was invented by Majid et al., based on the fluorescence resonance energy transfer (FRET) between a quantum dot (QD) as a donor and graphene oxide (GO) as an acceptor. When the target diazinon was added to the bioconjugates containing GO, photoluminescence recovery appeared to be the result of GO being detached from the aptamer due to the aptamer’s different affinity for GO. The biosensor’s detection limit was 0.13 nM, and the linearity ranged from 1.05 to 206 nM [45]. Yawen et al. developed a fast and accurate fluorescence resonance energy transfer (FRET) approach for the detection of diazinon in food at low concentrations. Graphene oxide (GO) was coupled with the synthesized aptamer-modified up conversion nanoparticles (Apt-UCNPs) via a π–π interaction. The fluorescence was diminished on the FRET between the UCNPs and GO. The aptamer formed a strong preference for binding with diazinon, which separated GO and improved the fluorescence signal. Under ideal circumstances, a limit of detection (LOD) of 0.023 ng/mL was attained with a broad linear detection range of 0.05 to 500 ng/mL [44]. The performance of nanobiosensors for the rapid and precise detection of pesticides can be considerably enhanced by the introduction of nanomaterials, particularly carbon quantum dots (CDs). In order to create a fluorescent biosensor, Maria et al. designed a naturally fluorescence and nontoxic CD coupled with acetylcholinesterase (AChE) as a bioreceptor. The biosensor was tested for the ability to detect profenofos, pure chlorpyrifos, and a commercial formulation known as Lorsban. For chlorpyrifos and Lorsban, respectively, a limit of detection (LOD) of 0.14 and 2.05 ppb was attained [46]. Different types of GO/rGO nanocomposites for pesticide detection along with their LOD have been given in Table 2.

## 6. Conclusions

In this review, we have highlighted various GO/rGO nanocomposites and their features for designing various sensor systems. We have presented the recent developments in GO/rGO–based sensors. The numerous types of sensors work on diverse principals, such as electrochemistry and fluorescence. It is evident that the unique functional properties of graphene and its various derivatives, such as excellent electrical conductivity, increased mobility of electrons, tuneable optical properties, ambient temperature quantum Hall effect, a significant surface–to–volume ratio, strong mechanical properties, and easiness of functionalization, make them quite excellent candidates for the development of biosensors. 

Furthermore, for the on-site monitoring and evaluation of pesticide residues in agricultural products and surroundings, the GO–enhanced Raman spectroscopic approach presents potential practical applications. With a better sensing performance than that of the conventional methods, the GO/rGO–based sensors allow for the quantitative determination of biologically significant compounds, ranging from tiny molecules and ions to large macromolecules. Due to their relatively recent development, the majority of sensor systems are still in the proof-of-concept stage, but they have already shown considerable promise for both practical and commercial applications. Furthermore, we believe that additional developments in the chemistry of GO and its derivatives will speed up the creation of relevant biosensors in widespread use with more reliable performance in many biological applications. The development of intelligent sensors for on–site point–of–care diagnostic testing using graphene-based materials seems possible and is expected to be cost-effective.

## 7. Limitation and Future Perspectives

Although there has been a number of recent developments in the design of graphene-based hybrid nanocomposites for multiple biosensor applications, it has remained difficult to comprehend the functionalities of graphene, GO, and rGO. It is also imperative to understand how they will interact with surface-modified polymers or inorganic nanoparticles. Although GO has many oxygen functional groups on its surface, using these functional groups effectively has been challenging because of their amorphous structure. It is quite difficult to achieve controlled uniform deposition and assembly onto graphene surfaces, and thus surface stability remains a major bottleneck in the synthetic methods. To obtain precisely controlled synthesis and a thorough understanding of the structure–property connections of graphene nanomaterials, more research efforts concentrating on novel yet inexpensive synthetic techniques are required. 

Because of the supporting electrodes’ roughness and interactions between the GO/rGO and electrode substrate, the underlying supporting electrode material is frequently not investigated, and this has an impact on the electrochemical sensor. However, in general, if a sensor is going to be used in the field, research should be conducted using screen-printed electrodes because of their high repeatability and scale of economy, which enable the translation of research from the lab to the field.

In addition, GO/rGO offers a variety of functionalization strategies for the recognition of biosensor components, offering multifaceted advantages over conventional biosensing transducer materials. The creation of high-quality, defect-free graphene sheets and their application in the commercial production of biosensors are still in the early stages. For the manufacturing of graphene to be ramped up, this obstacle must be removed. Future research is anticipated to overcome these issues for the low-cost commercial manufacturing of graphene. We think that in the next few years, the market for conventional biosensor technology for health monitoring may undergo a significant change as a result of the additive manufacturing of graphene, the Internet of Things, and wearable technology.

Due to numerous advantages over the conventional electrochemical method for sensing the pollutants, GO/rGO–based microfluidic devices are gaining increasing attention. The development of electrochemical biosensors and immunosensors for the detection of biologically significant markers is greatly facilitated by the excellent electrochemical characteristics of GO-based nanomaterials and nanocomposites. With improved sensitivity and detection limits that can reach subpicomolar levels, devices are being designed that employ a microporous 3D architecture. This approach appears to be effective for the detection of several biomolecules in a single–chip device at a time. However further studies are needed to work on affordable, on-site devices for detecting and measuring various harmful pesticide residues in water samples. 

## Figures and Tables

**Table 1:** Electrochemical sensing of pesticides using GO/rGO-based nanocomposites.

Nanomaterial	Pesticide	Linear Range	Limit of Detection	Ref.
AChE–CS/3DG–CuO NFs/GCE	Malathion	3 pm–46.665 nm	0.93 pM	[35]
Gra–CS AChE	Dichlorvos	0.1–100,000 nM	54 pM	[36]
GCE/VS_2_QDs–GNP/CMWCNTs/DZBA	Diazinon	5 × 10^−14^ mol L^−1^ to 1.0 × 10^−8^ nmol L^−1^	1.1 × 10^−14^ mol L^−1^	[30]
MoTe_2_ NPs/RGO	Profenofos	10^−9^ g L^−1^ and 10^−2^ g L^−1^	3.3 × 10^−10^ g L^−1^	[37]
CIS/rGO	Chlorpyrifos	0.5–470 ng mL^−1^	0.023 ng mL^−1^	[38]
AuNPs/FcDr/rGO/GCE	Dichlorvos	0.45–281.4 μM	0.21 μM	[39]
Ag/rGO/CS	Carbaryl	1.0 × 10^−8^ to 1.0 μg mL^−1^	1.0 × 10^−9^ μg mL^−1^	[40]
CNFs/GO/CS–GO/SPCE	Chlorpyrifos	2.5 nM–1 μM	2.2 nM	[41]

**Table 2:** Fluorescence sensing of pesticides using GO/rGO nanocomposites.

Nanomaterial	Pesticide	Linear Range	Limit of Detection	Ref.
Aptamer–GO–PEG	Profenofos	0.5–100 ng/mL	0.21 ng/mL	[47]
DNA TWJ-assembled G–quadruplex	Acetamiprid	0–500 nM	5.73 nM	[43]
rGQDs–MWCNT–aptamer	Diazinon	4–31 nM	0.4 nM (0.1 μg/L)	[45]
GO–L–cysteine capped CdS QDs/DF20 aptamer	Diazinon	1.05 to 206 nM	0.13 nM	[42]
Apt–UCNPs–GO	Diazinon	0.05 to 500 ng/mL	0.023 ng/mL	[44]
AChE–Carbon dots–GO	Chlorpyrifos	1 and 25 ppb (2.8–71 nM)	2.05 ppb (5.84 nM)	[48]

## Data Availability

Not applicable.

## Abbreviations

GO	graphene oxide
rGO	reduced graphene oxide
SPR	surface plasmon resonance
FRET	fluorescence resonance energy transfer
GC	gas chromatography
HPLC	high-performance liquid chromatography
CFR	Code of Federal Regulations
FAO	Food and Agriculture Organization
WHO	World health organization
OCPs	organochlorine substances
DDT	dichlorodiphenyltrichloroethane
AChE	acetylcholinesterase
PYR	pyrethroids
OP	organophosphates
MePO	methyl-paraoxon
0D	zero-dimensional
1D	one-dimensional
GNs	graphene nanosheets
TMDs	transition metal dichalcogenides
CNTs-	carbon nanotubes
LOD	limit of detection
LDI-MS	laser desorption and ionization mass spectrometry
SERS	surface-enhanced Raman spectroscopy
EIS	electrochemical impedance spectroscopy
CV	cyclic voltameter
MP	methyl parathion
QD	quantum dot
CD	carbon quantum dot
